# Cases report: MRI findings of asymptomatically familial subependymal heterotopia with filamin A gene abnormality

**DOI:** 10.3389/fnins.2022.956545

**Published:** 2022-07-27

**Authors:** Bin Lv, Yushan Zhou, Jianguang Zeng, Ling Wang, Fumin Zhao, Huizhu Chen, Xuesheng Li, Yu Song, Mei Xiao, Zhiyong Ding, Bochao Cheng

**Affiliations:** ^1^Department of Gynecology and Obstetrics, West China Second University Hospital, Sichuan University, Chengdu, China; ^2^Department of Nuclear Medicine, West China Hospital of Sichuan University, Chengdu, China; ^3^School of Economics and Business Administration, Chongqing University, Chongqing, China; ^4^Department of Ultrasound, West China Second University Hospital, Sichuan University, Chengdu, China; ^5^Department of Radiology, West China Second University Hospital, Sichuan University, Chengdu, China; ^6^Department of Medical Imaging, Qujing Maternal and Child Health Care Hospital, Kunming University of Science and Technology, Qujing, China; ^7^Key Laboratory of Birth Defects and Related Diseases of Women and Children, Sichuan University, Ministry of Education, Chengdu, China

**Keywords:** subependymal heterotopia, asymptomatic, magnetic resonance imaging, filamin A, case report

## Abstract

Subependymal heterotopia (SEH) is a rare neuronal migration disorder consisting of gray matter nodules along the lateral ventricular walls and is often associated with other brain malformations. Despite most SEH cases showing epilepsy during their lifetimes, very few patients with asymptomatically familial SEH tend to cause misdiagnosis or missed diagnosis. We present four familial SEH cases without any positive symptoms and medical history, including two fetuses, who were diagnosed by MRI and confirmed by genetic testing with mutation of filamin A. This report emphasizes the role of MRI in the recognition of SEH at an early age of gestation and in asymptomatically familial SEH. MRI provides a fast, repeatable, reliable, and cheap choice for detecting and screening familial SEH.

## Introduction

Subependymal heterotopia (SEH) is the most common subtype of Gray matter heterotopia (GMH) characterized by nodules of gray matter along the lateral ventricular walls (Zając-Mnich et al., 2014). GMH can be seen anywhere from ependyma to pia mater and has been clinically and morphologically divided into three types: SEH, subcortical heterotopia, and subcortical band heterotopia (Guerrini and Marini, 2006). SEH is the most common subtype characterized by nodules of gray matter along the lateral ventricular walls (Zając-Mnich et al., 2014). The main symptoms include epileptic seizures, cognitive decline, and neurological impairment (Zając-Mnich et al., 2014). Mutation of filamin A (FLNA) was reported in all cases with familial inheritance and around 25% of cases occurred sporadically (Zając-Mnich et al., 2014). Despite most cases having been reported to experience epilepsy during their lifetimes (Barkovich and Kuzniecky, 2000), SEH tends to be missed diagnosis and misdiagnosed due to small lesions and atypical symptoms. We present a group of familial inheritance SEH cases without any positive symptoms and medical history, including two fetuses, the pregnant woman, and her mother, who was diagnosed by MRI and confirmed by genetic testing. This report highlights the importance of MRI in early detection and quick screening of possible familial inheritance SEH.

## Case description

**FIGURE 1:**
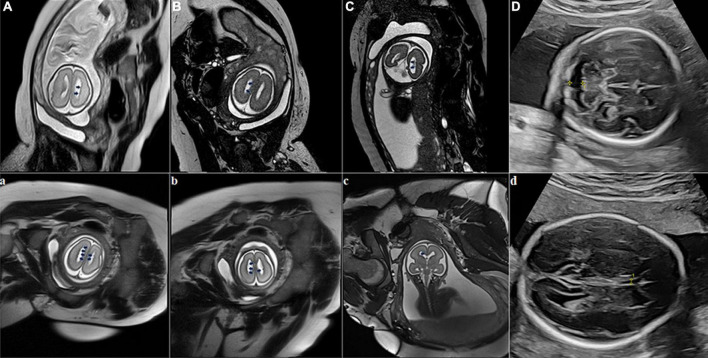
Fetal brain MRI at two fetuses of the pregnant woman. Fetus 1 **(A–D)**, scanning at 27 + 1 weeks of gestation with bilateral subependymal heterotopia (SEH). Axial (1A, 1B) and coronal (1C) fetal MRI images show that bilateral ventricles are not dilated, ependyma is irregular, and multiple abnormal signal nodules can be seen. The T2-weighted sequence shows nodules in the ventricular wall similar to gray matter signals (black arrows). Fetal sonogram **(D)** Axial image, obtained at 26 weeks of gestation through the cerebellar level, shows the widened posterior cranial fossa (yellow measurement arrow); Fetus 2 **(a–d)**, scanning at 25 + 2 weeks of gestation with bilateral SEH. Axial (1a, 1b) and coronal (1c) T2-weighted sequences at the level of the lateral ventricles show nodules in the ventricular wall similar to gray matter signals (black arrows). Fetal sonogram (1d) Axial image, obtained at 24 weeks of gestation through the double top diameter layer, shows the narrowing of the transparent compartment cavity (yellow measurement arrow).

A 34-year-old primigravid woman had an uneventful pregnancy until 26 weeks of gestation when she was admitted to our hospital due to abnormalities detected during fetal ultrasonography (FUS) examination. FUS suggested a widened posterior cranial fossa (Figure 1D), and maternal serum TORCH screening was negative. No positive symptoms including epilepsy, intellectual disability or dysplasia, abnormal physical examination, and positive family history (seizure, cognitive or motor deficits; adverse pregnancy and childbirth; developmental milestones, school performance, etc.) were observed concerning this pregnant woman, her husband, and their blood relatives. At 27 + 1 weeks of gestation, the woman accepted a fetal MRI without sedation on a 3-Tesla unit (Skyra, Siemens, Erlangen, Germany) using a multi-channel phased-array coil to allow increased coverage of the fetal head and increased signal-to-noise ratio. T2-HASTE, Trufi, T1-starvibe, and diffusion-weighted images (DWI) were acquired in planes that were approximately sagittal, coronal, and axial to the head of the fetus. The supine position, feet first, has been used throughout the examination to minimize fetal movements. The fetal had a widened posterior fossa cistern; irregular ependyma of bilateral lateral ventricles, and multiple ependymal multiple gray matter signal nodules (Figures 1A–C). Doctors suggested genetic testing, but the couple gave up the examination for price reasons and chose abortion.

**FIGURE 2:**
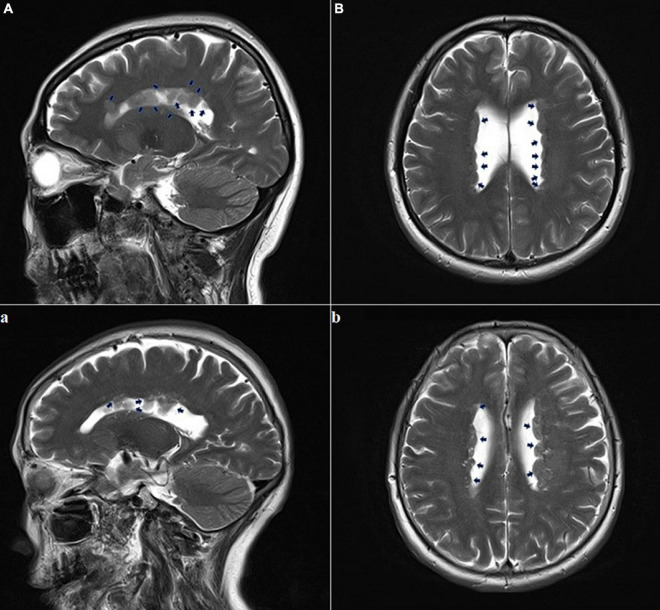
Adult brain MRI images of the pregnant woman and her mother. The pregnant woman **(A,B)**: T2-weighted sequence performed at the age of 35 years showed multiple foci of heterotopic gray matter outlining the walls of both lateral ventricles; this is consistent with the diagnosis of subependymal gray matter heterotopia (black arrows); The pregnant woman’s mother **(a,b)**: T2-weighted sequence performed at 67 years of age showing multiple foci of heterotopic gray matter outlining both lateral ventricles walls (black arrows).

A year later, the woman got pregnant again. At 24 gestational weeks, FUS showed a narrowing cavum septum pellucidum of the fetus (Figure 1d). Fetal MRI was performed at 25 + 2 gestational weeks using the same protocol as the first fetus and found multiple ependymal multiple gray matter signal nodules again (Figures 1a–c). Based on the above findings, SEH was suspected. The pregnant woman, her husband, and their blood relatives then accepted head MRI and electroencephalography (EEG). Head MRI was performed with the same 3-Tesla unit, and EEG was carried out using the international 10–20 system. T1-weighted image (T1WI), T2-weighted image (T2WI), fluid-attenuated inversion recovery (FLAIR) in the transverse, sagittal and frontal plane, and DWI in the transverse plane were required. MRI results suggested the pregnant woman and her mother had a mild expansion of the lateral ventricle, multiple ependymal multiple gray matter signal nodules, and a large occipital cistern (Figures 2A,B,a,b). The MRI results of other people and all the EEG results were negative. Further genetic examination revealed the abnormality of the FLNA gene c.7771dup (mother source) in the second fetus, the pregnant woman, and her mother.

## Discussion

Subependymal heterotopia showed a strong association with other structural anomalies including Chiari malformation, cerebral cortical malformations, callosal anomalies, etc. (Zając-Mnich et al., 2014). When SEH occurs alone without complications, patients normally have early development and come to medical attention with speech/psychomotor retardation, and the onset of epilepsy. Although the etiology of SEH is multi-factorial (Juric-Sekhar and Hevner, 2019), including genetic and epigenetic factors, the epigenetic mechanisms in the etiology of SEH mainly include hemorrhages, physical and chemical factors, radiation, and thermal injuries. Genetic factors influence the proliferation and differentiation of cortical neural progenitor cells (Watrin et al., 2015). Identified pathogenic genes associated with SEH can be subdivided into X-linked and non-X-linked inheritance patterns. Most cases are X-linked, frequently with the responsible FLNA, localized to Xq28 (Juric-Sekhar and Hevner, 2019). The product of the FLNA gene is a phosphoprotein-inducing actin reorganization and a mutation induces failure of the neuronal motility causing the premature arrest of neuronal migration (Fox et al., 1998). The gene product is essential for cell migration, and also important in the vascular and immune systems. SEH female persons usually have normal or mild cognitive decline, with epilepsy. For males, the condition often causes embryonic death. Very few surviving male patients have more severe neurologic and intellectual disabilities than female patients. Less frequent mutation in SEH concerns the ARFGEF2 gene (Ferland et al., 2009), 1p36 (Saito et al., 2008), 7q11.23 (Ferland et al., 2006), 5p15.1 (de Wit et al., 2009), etc. However, genetic testing is costly and time-consuming.

MRI is excellent for the detection and characterization of SEH nodules (Manganaro et al., 2009). The emergence of the fast scanning sequence of fetal MRI has overcome the difficulties with fetal movement, especially for small fetuses in early pregnancy. For instance, T2-weighted HASTE sequences allow multiple images to be acquired in a single maternal breath-hold and because of the high resolution and short scanning time, are the best imaging sequences for fetal MRI. A previous study has stated that fetal MR imaging is 40% sensitive and 50% specific in the diagnosis of SEH (Triulzi et al., 2011). However, the missed diagnosis rate is relatively high due to the poor imaging quality of some fetuses.

In our cases, the pregnant woman, her mother, and family members have no clinical abnormalities or other underlying etiology, which is consistent with the previous reports that some patients with SEH are neurologically and developmentally normal (Barkovich and Kuzniecky, 2000; Rojas et al., 2006). The negative symptoms induce clinicians’ neglect of familial inheritance. Although FUS is a safe method for the diagnosis of many fetal cerebral abnormalities, its application is limited in displaying brain parenchyma and the side close to the maternal spine and is susceptible to skull and gas interference, which leads to hard detection of SEH in early gestation. In addition, genetic testing is costly in developing countries and takes a long time to test. The adult MRI or fetal MRI provides a fast, repeatable, reliable, and cheap choice for detecting and screening familial SEH. We suggest that MRI or fetal MRI screening should be conducted on all family members in suspected cases, and MRI is the first choice when SEH is suspected.

## Data availability statement

The raw data supporting the conclusions of this article will be made available by the authors, without undue reservation.

## Ethics statement

This study was reviewed and approved by the local ethics committee of West China Hospital of Sichuan University. Written informed consent was obtained from the individual(s) for the publication of any potentially identifiable images or data included in this article.

## Author contributions

BL and BC: designing and writing the original draft. BC, JZ, and YZ: substantively revising the manuscript. LW, FZ, HC, XL, YS, MX, and ZD: data collection. BC: reviewing the final manuscript. All authors have read and approved the manuscript.

## Conflict of interest

The authors declare that the research was conducted in the absence of any commercial or financial relationships that could be construed as a potential conflict of interest. The handling editor JW declared a shared affiliation with the authors BC, ZD, and MX at the time of review.

## Publisher’s note

All claims expressed in this article are solely those of the authors and do not necessarily represent those of their affiliated organizations, or those of the publisher, the editors and the reviewers. Any product that may be evaluated in this article, or claim that may be made by its manufacturer, is not guaranteed or endorsed by the publisher.
